# Measuring, comparing and interpreting phenotypic selection on floral scent

**DOI:** 10.1111/jeb.14103

**Published:** 2022-09-30

**Authors:** Øystein H. Opedal, Karin Gross, Elodie Chapurlat, Amy Parachnowitsch, Nina Joffard, Nina Sletvold, Otso Ovaskainen, Magne Friberg

**Affiliations:** ^1^ Biodiversity Unit, Department of Biology Lund University Lund Sweden; ^2^ Department of Environment & Biodiversity Paris Lodron University of Salzburg Salzburg Austria; ^3^ Plant Ecology and Evolution, Department of Ecology and Genetics, EBC Uppsala University Uppsala Sweden; ^4^ Department of Ecology Swedish University of Agricultural Sciences Uppsala Sweden; ^5^ Department of Biology University of New Brunswick Fredericton New Brunswick Canada; ^6^ University of Lille, UMR 8198 – Evo‐Eco‐Paleo Lille France; ^7^ Department of Biological and Environmental Science University of Jyväskylä Jyväskylä Finland; ^8^ Organismal and Evolutionary Biology Research Programme University of Helsinki Helsinki Finland; ^9^ Centre for Biodiversity Dynamics, Department of Biology Norwegian University of Science and Technology Trondheim Norway

**Keywords:** floral fragrance, floral scent, natural selection, plant–pollinator interactions, reduced‐rank regression, selection gradient

## Abstract

Natural selection on floral scent composition is a key element of the hypothesis that pollinators and other floral visitors drive scent evolution. The measure of such selection is complicated by the high‐dimensional nature of floral scent data and uncertainty about the cognitive processes involved in scent‐mediated communication. We use dimension reduction through reduced‐rank regression to jointly estimate a scent composite trait under selection and the strength of selection acting on this trait. To assess and compare variation in selection on scent across species, time and space, we reanalyse 22 datasets on six species from four previous studies. The results agreed qualitatively with previous analyses in terms of identifying populations and scent compounds subject to stronger selection but also allowed us to evaluate and compare the strength of selection on scent across studies. Doing so revealed that selection on floral scent was highly variable, and overall about as common and as strong as selection on other phenotypic traits involved in pollinator attraction or pollen transfer. These results are consistent with an important role of floral scent in pollinator attraction. Our approach should be useful for further studies of plant–animal communication and for studies of selection on other high‐dimensional phenotypes. In particular, our approach will be useful for studies of pollinator‐mediated selection on complex scent blends comprising many volatiles, and when no prior information on the physiological responses of pollinators to scent compounds is available.

## Graphical abstract

Graphical summary of the reduced‐rank regression approach to estimating phenotypic selection on floral scent composition. The method reduces complex scent chemistry into a scent composite trait under selection (i.e. that is most strongly associated with variation in fitness), and estimates the strength of selection on this composite trait while also considering other phenotypic traits expected to be under selection.

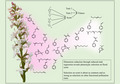

## 1 INTRODUCTION

The astonishing diversity of animal‐pollinated flowers is generally interpreted in light of adaptation to specific pollinators (Darwin, 1862; Fenster et al., 2004; Grant & Grant, 1965; Harder & Johnson, 2009; Stebbins, 1974). This hypothesis has spurred substantial interest in measuring pollinator‐mediated phenotypic selection on plant phenotypes (reviewed in Harder & Johnson, 2009, Caruso et al., 2019, Sletvold, 2019, Opedal, 2021). The measurement of selection on a limited set of well‐defined floral characters is statistically straightforward using the multiple‐regression approach of Lande and Arnold (1983). However, some functionally important floral phenotypes are not easily quantified through a small set of measurements. One important example is that of floral fragrances, which often comprise numerous volatile compounds (e.g. Friberg et al., 2019; Gfrerer et al., 2021; Raguso, 2008).

Recent insights into the biology of floral scent suggest that the scent bouquet should be a target of pollinator‐mediated phenotypic selection. First, floral scent is often variable at every level, that is among populations (Friberg et al., 2019; Parachnowitsch et al., 2012; Petrén et al., 2021), among individuals within populations (Friberg et al., 2017, 2019; Parachnowitsch et al., 2012; Zu et al., 2016) and within individuals (Burdon et al., 2015; Chapurlat et al., 2018; Friberg et al., 2014; Goodrich et al., 2006; Jürgens et al., 2014; Morinaga et al., 2008; Raguso & Weiss, 2015; Theis et al., 2007). Second, although more than 1000 volatile compounds have been detected in floral fragrances, the floral scent bouquets often comprise a core set of compounds of known biosynthetic background (Knudsen et al., 2006). Third, species divergence in scent chemistry is at least partly driven by pollinators, because distantly related species that share the same type of pollinator often exhibit similar floral scent chemistry (Dobson, 2006; Fenster et al., 2004; Junker & Parachnowitsch, 2015; Schiestl & Johnson, 2013; Whitten et al., 1986), whereas closely related species that interact with different pollinators often differ markedly in scent chemistry (Byers et al., 2014; Dobson et al., 1997; Hetherington‐Rauth & Ramírez, 2016; Weber et al., 2018).

Studies that have estimated selection on floral scent have often detected directional selection on the emission rate of one or more compounds (Chapurlat et al., 2019; Ehrlén et al., 2012; Gfrerer et al., 2021; Gross et al., 2016; Joffard et al., 2020; Parachnowitsch et al., 2012; Schiestl et al., 2010). However, studies of selection on floral scent are complicated both by our yet limited understanding of the functional role of floral scent in plant–pollinator communication (Schiestl, 2015) and by the high‐dimensional nature of floral fragrances, which create challenges for measuring selection (Chapurlat et al., 2019; Gfrerer et al., 2021; Gross et al., 2016; Parachnowitsch et al., 2012; Schiestl et al., 2010).

Biologically, the interpretation of selection estimates on floral scent is complicated by uncertainty about the extent to which pollinators are actively searching for certain compounds, or whether the scent of a flower as perceived by pollinators and other interactors (e.g. antagonists) is determined by the relative abundances of some or all of these compounds. There are examples of both strategies, but most studies come from highly specialized pollination systems which may not be representative of the behaviour of many pollinators. For example, plants can mimic insect alarm (Brodmann et al., 2009) or sex pheromones (e.g. Borg‐Karlson, 1990; Kullenberg & Bergström, 1976; Schiestl et al., 2003) that lure particular insect pollinators to the flowers. The compounds involved in these deceptive pollination systems are often unique, and not commonly part of floral scent blends. Similarly, plants involved in obligate pollination mutualism have sometimes evolved the release of particular compounds that function as ‘private channels’ to their particular mutualist species (Chen et al., 2009; Schäffler et al., 2015). In other specialized pollination mutualisms, plants emit diverse and generic floral scent compounds (Friberg et al., 2014, 2019; Ramírez et al., 2011), and their specialized pollinators have antennal receptors that detect several to many of these volatiles (Eltz & Lunau, 2005; Schiestl et al., 2021; Svensson et al., 2010). To further complicate the issue, many flowering plants are pollinated by generalist insects (Johnson & Steiner, 2000; Waser et al., 1996), and these are able to learn different floral scents, singularly or in blends (Lawson et al., 2018; Riffell et al., 2008; Wright et al., 2013; Wright & Schiestl, 2009). In the latter cases, the trait ‘scent’ may represent a combination of a potentially large number of measurements (volatile concentrations), and it is unclear how pollinators use the multidimensionality of floral scent variation in their interaction with flowers (García et al., 2021; Wright & Schiestl, 2009). Hence, analyses of selection on scent need to consider both individual floral scent compounds and the entire scent bouquet (as a ‘composite trait’).

Studies of selection on scent are also complicated statistically by high dimensionality and associated issues related to multicollinearity (Graham, 2003). The most common solution to the problem of measuring selection on high‐dimensional phenotypes is to employ dimension reduction through principal component regression (Gross et al., 2016; Parachnowitsch et al., 2012; Schiestl et al., 2010). In this two‐step approach, dimension reduction is achieved by projecting an original set of covariates (volatile concentrations) onto a subset of principal components, which are subsequently included as predictors in a multiple‐regression model. This approach solves the issue of fitting regression models to high‐dimensional data but yields estimates of selection that are not directly linked to the original trait measurements (but see Chong et al., 2018).

The aim of dimension reduction in principal component regression is to reduce the multivariate phenotype into a subset of phenotypic axes that jointly explain most of the variance in the original phenotypic space. In other words, dimension reduction for the phenotype is performed independently of the relationship between phenotype and fitness. This is potentially problematic because the most variable phenotypic axes may not be those that are ecologically most important or interesting (Morrissey, 2014; Schluter & Nychka, 1994). An alternative approach to dimension reduction is to explicitly seek the phenotypic axes (combinations of the original variables) that explain the most variance in the response variable (e.g. relative fitness). This can be achieved through techniques such as two‐block partial least‐squares (Gómez et al., 2006; Rohlf & Corti, 2000), projection‐pursuit regression (Friedman & Stuetzle, 1981; Morrissey, 2014; Schluter & Nychka, 1994) or reduced‐rank regression (Anderson, 1951). These approaches allow estimating the leading axes of phenotypic variation that are under selection, a very useful property for analyses of multivariate selection (Morrissey, 2014). In turn, selection gradients on the original traits can be obtained via numerical methods (Morrissey & Sakrejda, 2013), or by projecting the estimated selection on the leading axes back to the original trait space as suggested for principal component regression (Chong et al., 2018). This facilitates biological interpretation in cases where dimension reduction is applied for traits with a clear functional role in the process under study (e.g. floral dimensions in studies of pollinator‐mediated selection; Opedal, 2021) and may also be helpful for characterizing and interpreting the structure of the major axes of selection in cases where the biological relevant phenotype represents a combination of the original measurements.

The aim of this study is to reassess general patterns of phenotypic selection on floral scent through a re‐analysis of data from four previously published studies (Chapurlat et al., 2019; Gross et al., 2016; Joffard et al., 2020; Parachnowitsch et al., 2012). We use Bayesian reduced‐rank regression to jointly estimate the major axis of selection on floral scent and the strength of selection acting on this axis as well as additional morphological and phenological traits. Specifically, we ask: (1) How well can selection on floral scent be characterized by reducing variation in floral scent into a single ‘scent selection axis’? (2) How strong is phenotypic selection on floral scent (as a composite trait)? (3) Does selection on floral scent vary among species, over time and across space? We further discuss and demonstrate how estimated selection on scent as a composite trait can be translated back to the original scent variables, thus facilitating interpretation.

## 2 MATERIALS AND METHODS

### 2.1 Theory: phenotypic‐selection analysis with reduced‐rank regression

Reduced‐rank regression (Anderson, 1951; Izenman, 1975) achieves dimension reduction in multivariate problems by projecting an original set of covariates onto a reduced set of composite variables that best explains variance in the response variable. In selection analysis, this translates into the reduced set of phenotype axes that best explains relative fitness and, thus, is under selection. In the following analyses, we used the Bayesian reduced‐rank regression implementation of the Hmsc 3.0 R package (Ovaskainen & Abrego, 2020; Tikhonov et al., 2020).

In the Hmsc model, the linear predictor for the fixed effects is written as $L_{\mathrm{ij}}^{F}=\sum_{k}x_{\mathrm{ik}}\beta_{\mathrm{kj}}$, where $x_{\mathrm{ik}}$ is the value of covariate *k* for observation *i*, and $\beta_{\mathrm{kj}}$ is the regression slope of response variable j on covariate k. In the following analyses, we include only one response variable, but we keep the multivariate notation here for generality. In the reduced‐rank regression implementation, the $n_{c}$ covariates k are decomposed into two sets so that $n_{c}=n_{c}^{*}+n_{c}^{\mathrm{RRR}}$. The covariates $k=1,\ldots ,n_{c}^{*}$ are treated as standard regression covariates, while dimension reduction is applied for the covariates $k=\left(n_{c}^{*}+1\right),\ldots ,\left(n_{c}^{*}+n_{c}^{\mathrm{RRR}}\right)$. The number of original covariates for which dimension reduction is applied is denoted $n_{c}^{O,\mathrm{RRR}}$, and the number of resulting covariates $n_{c}^{\mathrm{RRR}}$. The reduced‐rank regression covariates are obtained as linear combinations of the original covariates, $x_{i\left(n_{c}^{*}+k\right)}=\sum_{l=1}^{n_{c}^{O,\mathrm{RRR}}}w_{\mathrm{kl}}{\tilde{x}}_{\mathrm{il}}$ (for $k=1,\ldots ,n_{c}^{\mathrm{RRR}}$), where the weights $w_{\mathrm{kl}}$ determine the contribution of the original covariates ${\tilde{x}}_{\mathrm{il}}$ to the new covariate $x_{i\left(n_{c}+k\right)}$. The weights $w_{\mathrm{kl}}$ and the regression coefficients $\beta_{\mathrm{kj}}$ are estimated during model fitting (posterior sampling). Note that this implementation allows us in selection analyses to separate phenotypic traits into a set of $n_{c}^{*}$ traits for which selection is estimated in the standard way, and a set of $n_{c}^{O,\mathrm{RRR}}$ traits for which dimension reduction is applied. This is relevant for studies of selection because we often want to estimate selection directly on certain traits such as flower number and flower size, while applying dimension reduction to composite traits such as scent represented by a large number of volatile concentrations.

When the response variable is relative fitness (individual absolute fitness divided by population‐mean fitness), the estimated regression slopes $\beta_{\mathrm{kj}}$, including those for the reduced‐rank covariates, provide estimates of selection gradients (i.e. partial derivatives of relative fitness with respect to phenotype). To understand how a given set of selection gradients on scent as a composite trait translates into selection on the original variables, we can project the selection estimates back onto the original variables as $\beta_{\mathrm{lj}}^{*}=\sum_{k}w_{\mathrm{kl}}\beta_{\left(n_{c}^{*}+k\right)j}$. In case of a single response variable, the resulting column vector $\beta_{l1}^{*}$ contains the selection estimates on the $n_{c}^{O,\mathrm{RRR}}$ original covariates. This approach is directly analogous to the approach proposed by Chong et al. (2018) for principal component regression.

### 2.2 Study systems

We analysed 22 datasets (population‐year combinations) compiled from four previous studies. These include one population of *Gymnadenia conopsea* (Orchidaceae) from Sweden (Chapurlat et al., 2019), eight populations of *Gymnadenia odoratissima* from Switzerland, five of which were studied in 2 years (Gross et al., 2016), seven populations belonging to three subspecies of *Anacamptis coriophora* (Orchidaceae) from France (Joffard et al., 2020) and one population of *Penstemon digitalis* (Plantaginaceae) from Canada (Parachnowitsch et al., 2012). As a case study of spatio‐temporal variation in selection on floral scent, we focused on *Gymnadenia odoratissima*. Four of the eight study populations were located in the lowland and four in the mountains. Of these, three lowland populations and two mountain populations were studied in 2 years. The phenotypic data include three morphological traits (flower number, plant height and inflorescence length) and 22 floral volatiles. Further details about all study systems and study designs are given in the Appendix S1.

### 2.3 Selection analyses

We analysed each of the 22 datasets (population‐year combinations) separately and refer to these as ‘studies’. In all analyses, individual plants were treated as sampling units, and female reproductive success (fruit production) as a fitness proxy. All datasets included abundances of scent compounds (volatiles hereafter) as well as morphological traits, and some included a phenological trait (flowering time).

We fitted Hmsc models to each dataset with relative fitness as response variable and Gaussian error distribution. As fixed effects, we included the morphological and phenological traits as ‘standard’ covariates (specified by the XData argument in Hmsc), while the volatiles were reduced into a single ‘scent selection axis’ through reduced‐rank regression specified through the XRRRData argument in Hmsc. The models did not include any random effect. The R code implementing all analyses is available on GitHub; github.com/oysteiop/ScentSelection).

We obtained mean‐ (*β*_μ_) and variance‐scaled (*β*_σ_) linear selection gradients for the standard traits and the scent selection axis by multiplying the regression slope on each covariate by its mean and standard deviation, respectively (Hereford et al., 2004). Because the scent selection axis is not on a ratio scale, mean‐scaling is not meaningful (Hereford et al., 2004; Houle et al., 2011) and we report only variance‐scaled selection gradients for the scent selection axis. After projecting the estimated selection gradient on the scent selection axis back onto the original volatiles to facilitate interpretation, we expressed inferred selection on each volatile as mean‐scaled selection gradients.

To evaluate the adequacy of the dimension reduction approach for characterizing selection on floral scent, we compared the explanatory and predictive power of the reduced‐rank regression models to models treating each volatile concentration as a standard covariate (Lande & Arnold, 1983). To compare the predictive power of the two models (i.e. reduced‐rank regression for the volatiles vs. standard multiple‐regression for all traits), we performed fivefold cross‐validation in which we split the data into five ‘folds’ and sequentially obtained predictions for each fold from a model trained on the four remaining folds. We then computed predictive *r* ^2^‐values as the squared correlation between the predicted and observed values.

For *G. odoratissima*, we assessed spatio‐temporal variation in selection on each compound through the approach of Albertsen et al. (2021), in which the among‐dataset variation is computed as

$$\sigma_{\beta }^{c}=\sqrt{\sigma_{\beta }^{2}-\bar{{\mathrm{SE}}_{\beta }^{2}}},$$

where $\sigma_{\beta }^{c}$ is the variance of the selection‐gradient estimates among datasets, and ${\mathrm{SE}}_{\beta }^{2}$ is the sampling variance of each selection‐gradient estimate. In the current Bayesian framework, we used the variance of the posterior distribution as an estimate of the sampling variance (squared standard error). For mean‐scaled selection gradients, this measure can be interpreted as the mean dispersion of the selection estimates in units of the strength of selection on fitness itself.

## 3 RESULTS

On average across all 22 studies, one standard deviation change in floral scent (as a composite trait) changed relative fitness by 15.4% (mean *β*_scent_ = 0.154, median = 0.063, range = 0.001–0.528, Table 1). Selection on scent was well supported statistically (posterior support >90%) in about 41% of the studies (9/22 studies). In the remaining 13 studies, support for selection was weak to moderate (posterior support 50.6%–78.0%).

**TABLE 1:** Summary of selection estimates (variance‐scaled selection gradients, |*β*|_scent_) on floral scent as a composite trait with 95% credible intervals

Species	Population: Year	*n*	*n*_vol_	|*β*|_scent_ (95% CI)	P[|*β*|_scent_ >0]	*r* ^2^	$r_{\mathrm{MR}}^{2}$	$r_{\mathrm{CV}}^{2}$	$r_{\mathrm{CV}‐\mathrm{MR}}^{2}$	*r*_β_
*Gymnadenia conopsea*	Folkeslunda: 2016	169	14	**0.066 (−0.050, 0.145)**	**0.908**	70.2%	72.4%	65.6%	59.8%	0.89
*Gymnadenia odoratissima*	Döttingen (lowland): 2010	73	22	**0.362 (0.039, 0.634)**	**0.984**	52.5%	70.0%	28.1%	17.9%	0.67
	Döttingen (lowland): 2011	92	22	**0.498 (0.189, 0.744)**	**0.990**	45.4%	56.0%	24.7%	13.9%	0.74
	Linn (lowland): 2010	92	22	0.078 (−0.204, 0.327)	0.720	35.9%	47.2%	21.0%	5.9%	0.47
	Linn (lowland): 2011	92	22	0.066 (−0.106, 0.227)	0.780	50.6%	66.0%	39.4%	10.9%	0.67
	Remigen (lowland): 2010	88	22	**0.343 (−0.181, 0.716)**	**0.922**	34.3%	48.7%	6.4%	6.7%	0.71
	Remigen (lowland): 2011	56	22	**0.235 (−0.050, 0.483)**	**0.938**	50.2%	77.2%	24.2%	39.8%	0.51
	Rossweid (lowland): 2011	72	22	0.052 (−0.127, 0.212)	0.732	55.3%	75.4%	48.6%	23.4%	0.73
	Albulapass (mountain): 2010	69	22	0.053 (−0.220, 0.321)	0.637	46.0%	59.4%	35.4%	9.2%	0.73
	Corviglia (mountain): 2011	82	22	0.037 (−0.13, 0.206)	0.658	45.2%	63.0%	38.1%	21.6%	0.70
	Münstertal (mountain): 2010	96	22	0.058 (−0.109, 0.22)	0.746	53.8%	67.9%	49.1%	32.8%	0.77
	Münstertal (mountain): 2011	94	22	**0.120 (−0.085, 0.267)**	**0.903**	66.6%	76.5%	54.3%	55.6%	0.84
	Schatzalp (mountain): 2010	47	22	**0.354 (−0.073, 0.653)**	**0.953**	62.2%	86.8%	21.8%	30.0%	0.66
	Schatzalp (mountain): 2011	75	22	0.059 (−0.146, 0.244)	0.730	29.8%	47.7%	17.4%	6.9%	0.67
*Anacamptis coriophora coriophora*	Camprieu: 2016	54	32	0.004 (−0.180, 0.179)	0.506	16.9%	65.3%	7.2%	12.0%	0.50
	Comps sur Artuby: 2016	48	27	**0.528 (0.074, 0.978)**	**0.982**	66.2%	80.7%	36.0%	18.9%	0.80
	Sournia: 2016	55	31	0.007 (−0.264, 0.278)	0.523	26.4%	71.2%	0.1%	5.5%	0.41
*Anacamptis c. fragrans*	Blandas: 2016	44	29	0.013 (−0.238, 0.271)	0.524	11.8%	56.1%	0.0%	0.1%	0.53
	Le Cannet des Masures: 2016	60	26	**0.357 (−0.161, 0.834)**	**0.940**	32.3%	53.8%	9.4%	8.6%	0.58
	Trassanel: 2016	54	30	0.001 (−0.241, 0.238)	0.512	15.0%	49.8%	1.4%	0.1%	0.59
*Anacamptis c. martrinii*	Saillagouse: 2016	54	32	0.054 (−0.227, 0.357)	0.659	11.3%	71.1%	0.8%	4.3%	0.66
*Penstemon digitalis*	Common garden: 2007	88	23	0.043 (−0.071, 0.166)	0.751	86.7%	93.8%	80.9%	73.1%	0.88
Mean				0.154		43.8%	66.2%	27.7%	20.8%	0.67
Median				0.063		45.7%	67.0%	24.5%	13.0%	0.67

*Note*: |*β*|_scent_ represents the strength of selection but is non‐directional and is given as an absolute value. Other parameters are sample size (*n*), number of volatiles (*n*_vol_), posterior support for selection on scent (P[|*β*|_scent_ >0]), explanatory power for the reduced‐rank regression model (*r* ^2^) and the multiple‐regression model ($r_{\mathrm{MR}}^{2}$), and predictive power based on fivefold cross‐validation for the reduced‐rank regression ($r_{\mathrm{CV}}^{2}$) and multiple‐regression models ($r_{\mathrm{CV}‐\mathrm{MR}}^{2}$). The column *r*_β_ gives the correlation between compound‐specific selection gradients inferred from the reduced‐rank regression and multiple‐regression models.

Bold values indicate at least 90% posterior support for selection on scent.

Explanatory power was always higher for the multiple‐regression models than for the reduced‐rank regression models (Table 1). When making predictions for independent training data (cross‐validation), however, the reduced‐rank regression models often performed as well or better than the multiple‐regression model (Table 1).

The compound‐specific selection estimates inferred by projecting selection on the scent selection axis back onto the original variables were qualitatively similar to those obtained through standard multiple regression, as indicated by moderate‐to‐strong positive correlations between selection gradients inferred by the two methods (mean *r* = 0.67, range = 0.41–0.89).

### 3.1 Spatio‐temporal variation in selection on scent in *G. odoratissima*

**FIGURE 1:**
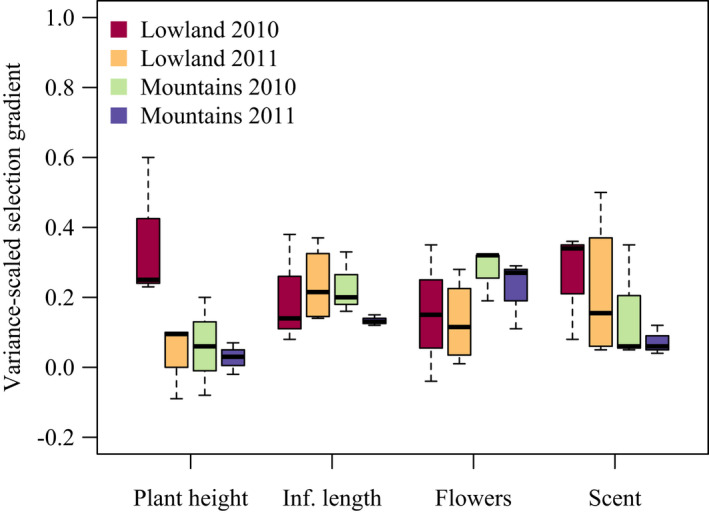
Variance‐scaled linear selection gradients on morphological traits (plant height, inflorescence length and number of flowers) and floral scent (a composite trait) across lowland and mountain populations of *Gymnadenia odoratissima* in Switzerland.

Selection on scent and other pollination traits (flower number, plant height and inflorescence length) of *G. odoratissima* varied in time and space and specifically tended to be stronger in the lowlands than in the mountains, especially in 2010 (Figure 1). Selection on scent was reasonably strong (*β*_scent_ >0.1) and statistically well supported in 6 of 13 studies (population‐year combinations, Table 1).

**FIGURE 2:**
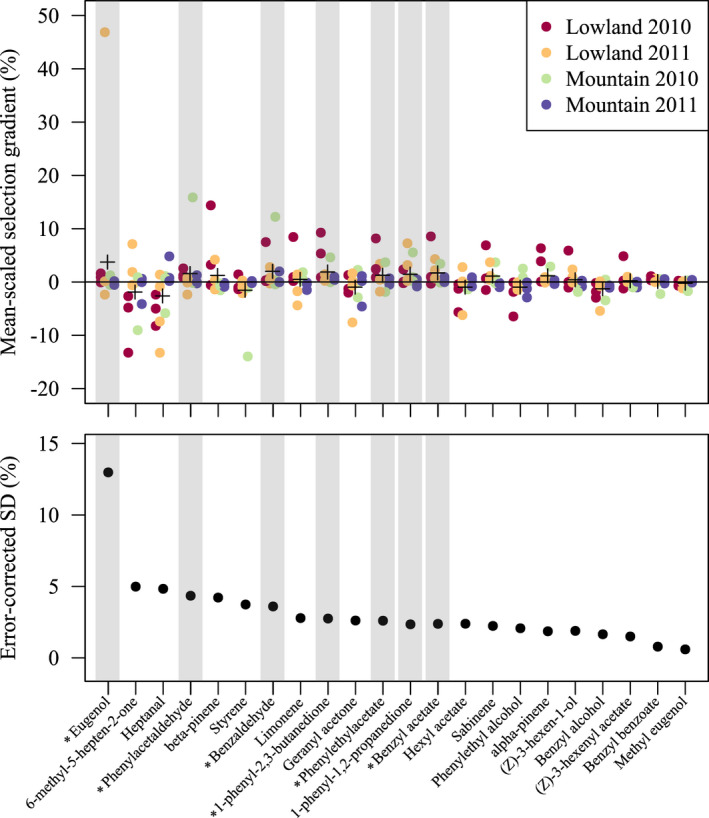
Spatio‐temporal variation in compound‐specific mean‐scaled linear selection gradients in *Gymnadenia odoratissima*. In the upper panel, the + indicates the mean for each compound. The lower panel shows the standard deviation of the selection gradients on each compound, after correcting for the sampling variance in the individual estimates. The grey bars indicate compounds that loaded onto the leading principal component in Gross et al. (2016). Gross et al. (2016) detected positive selection on PC1 and stronger selection in the lowlands than in the mountains. Asterisks (*) indicate compounds that were shown to be electrophysiologically active in pollinators

Inferred selection on individual volatiles also varied in time and space, yet the magnitude of variation was limited after accounting for sampling uncertainty (Figure 2). Notably, average selection gradients on all volatiles were close to zero.

## 4 DISCUSSION

Phenotypic selection on floral scent composition is implicit to the hypothesis that pollinators and other biotic interactors drive the evolution of floral scent. Although most of our study species are terrestrial orchids, and studies in other systems are needed to confirm their generality, our analyses yielded several novel insights into patterns of selection on floral scent. First, by leveraging dimension reduction through reduced‐rank regression, we have shown that selection on scent can often be well characterized by reducing variation in scent composition into a single axis of variation under selection. Second, the average selection intensity on scent as a composite trait (*β*_σ_ = 0.154, Table 1) is comparable to mean selection intensities on other traits involved in pollinator attraction or pollen transfer (‘pollination traits’, e.g. flower size, plant height, flower–pollinator‐fit traits; Harder & Johnson, 2009, Opedal, 2021). Third, the statistical support for selection on scent in about a third of the studies is also comparable to patterns observed for other kinds of pollination traits.

Field experiments (Chapurlat et al., 2019), experimental evolution (Gervasi & Schiestl, 2017) and analysis of trait–performance–fitness relationships cf. (Arnold, 1983; Opedal, 2021) suggest that pollinators are often the principal agents of selection on floral scent. While pollinator‐mediated selection on flower dimensions can often be interpreted trait by trait (Opedal, 2021), it is unclear whether selection on floral scent acts on individual volatiles or on the entire scent bouquet. Indeed, scent bouquets comprise sets of biochemically linked compounds (Junker et al., 2018), and scent chemistry should perhaps be seen as a reducible multivariate phenotype rather than as an irreducible multidimensional trait (Collyer et al., 2015). We found that the dimension reduction approach captured well the relationship between phenotype and fitness (i.e. selection), but this is not directly informative about how pollinators respond to variation in scent. To further understand the biological meaning of the ‘scent selection axis’ inferred by our approach, data are needed on how pollinators respond physiologically to compounds inferred to be under selection. There is ample evidence that pollinators respond physiologically to floral volatiles (e.g. Dötterl et al., 2006; Eltz & Lunau, 2005; Schiestl et al., 2021; Svensson et al., 2010) and that floral volatiles are attractive to pollinators in the field (Dodson et al., 1969; Majetic et al., 2009). To facilitate such functional studies, selection on scent as a composite trait can be readily translated into compound‐specific selection gradients using a method analogous to that proposed by Chong et al. (2018) for principal component regression. To assess the role of individual compounds vs. blends, the results could be used to produce synthetic mixtures of compounds representing volatile combinations inferred to be associated with high vs. low fitness and evaluate whether pollinators respond differently to single compounds vs. blends.

Our analyses of compound‐specific selection in Swiss *Gymnadenia odoratissima* populations (Gross et al., 2016) suggested that, while accounting for sampling uncertainty, selection on all compounds varied detectably in time and space. Interestingly, the mean selection gradient was close to zero for all compounds, suggesting that selection fluctuates both in strength and direction between years and among populations. Although floral scent is functionally involved in advertisement towards pollinators, these patterns of variation in selection are closer to those observed for pollinator‐fit traits than for other advertisement traits such as plant height or flower display size (Opedal, 2021). We can speculate that spatio‐temporal variation in selection on scent chemistry is driven by variation in pollinator assemblages, as seems often to be the case for fit traits (e.g. Chapurlat et al., 2015; Herrera et al., 2006; Opedal, 2021; Paudel et al., 2016; Soteras et al., 2020). While variation in selection on fit traits is expected to arise from variation in the fit of local pollinators to flowers, variation in selection on scent could well arise from variation in the scent preferences of local pollinators (Ramírez et al., 2011; Suinyuy et al., 2015). Further tests of this hypothesis could leverage, for example reciprocal‐transplant experiments or common‐garden studies with plants sourced from populations exhibiting distinct scent.

Previous studies of selection on floral scent have taken diverse approaches to overcome the high dimensionality of floral scent data. Chapurlat et al. (2019) reduced the dimensionality of the scent data by pre‐selecting a reduced set of compounds known to elicit physiological responses in the pollinator species observed at the study site, and by eliminating compounds causing correlation problems. For this dataset, the original analysis was practically identical to our multiple‐regression analysis, and the compound‐specific selection gradients so inferred were strongly correlated to those inferred by our reduced‐rank regression approach (*r* = 0.89, Table 1). Gross et al. (2016) and Joffard et al. (2020) chose instead to include all detectable volatile compounds and instead reduced dimensionality through principal component regression. Comparing results across studies is harder in these cases, but our results are qualitatively comparable to those of Gross et al. (2016) in that selection in scent tended to be stronger in lowland populations, especially in the first year of study. Furthermore, the analysis of compound‐specific selection was consistent with the results of Gross et al. (2016) in terms of which compounds were under stronger selection (Figure 2, and see Appendix S1). Our results are also qualitatively comparable to those of Joffard et al. (2020) in identifying the same two populations subject to stronger selection.

Reduced‐rank regression and principal component selection are not the only statistical techniques for dealing with large sets of correlated predictor variables. One possibility is to use regularization approaches such as the elastic net (Zou & Hastie, 2005) and its variants such as the least absolute shrinkage and selection operator (‘lasso’). Like our reduced‐rank regression approach, these approaches aim at maximizing the predictive ability rather than model fit (Morrissey, 2014). Gfrerer et al. (2021) used an elastic‐net approach in their recent study of *Arum maculatum*, a species with extraordinarily complex floral scent chemistry. These authors used the elastic‐net approach to identify which of the 289 compounds emitted by their study plants were more strongly associated with fitness and subsequently estimated selection on these compounds using standard multiple‐regression. Another suitable approach is projection‐pursuit regression as advocated by Schluter and Nychka (1994). This approach is similar to reduced‐rank regression, although allows non‐linearity in the functions used to construct the predictors (Morrissey, 2014). Given the difficulties involved in collecting scent data, and the modest sample sizes typically achievable, it is not clear that adding such complexity would yield much further insight. Finally, while not yet applied to studies of floral scent, morphometric studies have estimated selection on shape (as a multidimensional trait) through the two‐block partial least‐squared method, which also yields axes of maximum covariance between sets of variables such as fitness and shape (Gómez et al., 2006; Kuchta & Svensson, 2014; Rohlf & Corti, 2000).

All these approaches yield insights into patterns of selection on scent chemistry, although we argue that there are several advantages of reduced‐rank regression and similar approaches. First, comparison to published principal component regression analyses (Gross et al., 2016; Joffard et al., 2020) suggests that the two approaches to dimension reduction yield qualitatively similar conclusions, yet the numerical interpretability remains higher for the reduced‐rank regression approach due to the direct inference of the axis of scent variation under selection. Second, compound‐specific selection gradients inferred by multiple‐regression vs. reduced‐rank regression appears strongly correlated when the number of compounds is relatively low and the sample size is relatively large (Table 1). The advantage of the reduced‐rank regression approach is that we also obtain an estimate of ‘overall’ selection on scent, and the strength of selection on the scent composite trait was not obviously related to sample size or to the number of volatiles included in the analysis. Pre‐selecting compounds based on knowledge about pollinator responses are clearly biologically meaningful, but the downside of this approach is that data on physiological responses may often not be available, and it is not clear whether the physiological response to a compound maps directly to the relevance of these compounds in foraging decisions. Furthermore, analysing a subset of compounds with reduced collinearity, or that are found to be under stronger net selection, could bias inferred patterns of ‘overall’ selection on scent. Taken together, these points suggest that the reduced‐rank regression approach may be particularly useful for studies of selection on complex scent blends comprising many compounds, and when no prior information on physiological responses of pollinators is available.

Our reduced‐rank regression approach can be easily extended to accommodate different data types. The flexible Hmsc model allows analysing several response variables jointly, which provides interesting possibilities for studies of selection. First, selection studies sometimes consider several fitness components, such as pollinator visitation, pollen deposition, seed set and seeds sired through pollen export (male fitness). By including several of these fitness components as separate response variables, it is possible to ask how variation in floral scent affects each, while accounting for potential covariance among fitness components. Similarly, reproductive success of plants may depend not only on pollinator visitation, but, for example, also on seed predation (Parachnowitsch & Caruso, 2008; Pérez‐Barrales et al., 2013). When multiple response variables are included in the model, it also becomes natural to include multiple reduced‐rank covariates to allow for distinct patterns of response to floral scent for, say, pollinators and seed predators. Finally, we note that our approach could be directly applied to other high‐dimensional problems, such as those involved in measuring selection on chemical traits more generally (e.g. nectar or leaf defensive chemistry), or on shape quantified through morphometric methods (Gómez et al., 2006).

## 5 CONCLUSIONS

Our reduced‐rank regression approach allowed us to obtain a measure of selection on scent as a composite trait and, thus, to quantify the strength of selection on a scale allowing direct comparison to other trait types. These analyses yielded the novel insight that, in the taxa we studied, selection on scent is about as common and as strong as selection on other traits functionally involved in pollination. This result supports the hypothesis that scent‐mediated plant–pollinator interactions can drive floral evolution. Our analyses also suggest that dimension reduction can yield an adequate characterization of the floral scent fitness surface in many cases and underlines the importance of further studies combining estimates of selection on scent with functional studies of pollinator cognition. Our approach also facilitates this by identifying compounds under stronger selection, which can subsequently be included in functional studies of pollinator physiological responses.

## AUTHOR CONTRIBUTIONS

**Øystein H Opedal:** Conceptualization (lead); data curation (lead); formal analysis (lead); funding acquisition (lead); investigation (lead); methodology (lead); project administration (lead); software (lead); visualization (lead); writing – original draft (lead); writing – review and editing (lead). **Karin Gross:** Data curation (equal); investigation (equal); visualization (supporting); writing – original draft (supporting); writing – review and editing (supporting). **Elodie Chapurlat:** Data curation (supporting); investigation (supporting); writing – original draft (supporting); writing – review and editing (supporting). **Amy Parachnowitsch:** Conceptualization (supporting); data curation (supporting); investigation (supporting); writing – original draft (supporting); writing – review and editing (supporting). **Nina Joffard:** Data curation (supporting); investigation (supporting); writing – original draft (supporting); writing – review and editing (supporting). **Nina Sletvold:** Conceptualization (supporting); writing – original draft (supporting); writing – review and editing (supporting). **Otso Ovaskainen:** Formal analysis (supporting); software (supporting); writing – original draft (supporting); writing – review and editing (supporting). **Magne Friberg:** Conceptualization (supporting); writing – original draft (supporting); writing – review and editing (supporting).

## CONFLICT OF INTEREST

The authors declare no conflict of interest.

### PEER REVIEW

The peer review history for this article is available at https://publons.com/publon/10.1111/jeb.14103.

## Supporting information


Appendix S1:

## Data Availability

All data and R‐code are available in GitHub (github.com/oysteiop/ScentSelection), doi: 10.5281/zenodo.7085996.
