# Supplementary material for: Measuring, comparing and interpreting phenotypic selection on floral scent
Source: J Evol Biol. 2022 Sep 30;35(11):1432–41. doi: 10.1111/jeb.14103 (PMC9828191; doi:10.1111/jeb.14103)
Supplement: Supplementary file 1 — Appendix S1: [file JEB-35-1432-s001.docx]

**Measuring, comparing, and interpreting phenotypic selection on floral scent**

**SUPPLEMENTARY MATERIALS**

**Extended methods**

*Study systems*

*Gymnadenia conopsea* s.s.

Chapurlat *et al.* (2019) studied the fragrant orchid *Gymnadenia conopsea* s.s., a tuberous, nonclonal and long‐lived perennial (Øien & Moen, 2002). The fragrant flowers open sequentially from the bottom to the top of a single spike inflorescence of 10-100 flowers. Individual flowers remain open for up to one week, and individual plants may flower for a month. Each flower contains two pollinaria, which are situated above the spur entrance. Plants are self‐compatible but depend on pollinators for successful fruit set (Sletvold *et al.*, 2012). The study was conducted in summer 2016 in a population located at Folkeslunda on the island of Öland, southern Sweden (56° 43′N, 16° 44′E), where the most frequent pollinators were the nocturnal *Deilephila porcellus* and *Autographa gamma*, with rare diurnal visits by *Aglais urticae* and *A. gamma*. The phenotypic data include one phenological trait (date of first flowering), four morphological traits (flower number, plant height, corolla area, spur length), and 108 scent traits (concentrations of 54 volatile compounds measured during daytime and at night). The study plants were subjected to either a control (open-pollinated) treatment, or a hand-pollination treatment in which stigmas were saturated with pollen to reduce the opportunity for pollinator-mediated selection. We analysed the two treatments separately. To make the results comparable to the original analysis, we restricted the analyses to the subset of 14 scent traits considered in the original study. This subset comprised those compounds known to elicit antennal responses for the pollinator species observed in the population that season, that were present in at least 20% of the samples, and excluded three variables causing correlation problems in the original analysis.

*Gymnadenia odoratissima*

Gross *et al.* (2016) studied eight populations of the orchid *Gymnadenia odoratissima* in Switzerland. Four of these populations were located in the lowland and four in the mountains. Of these, three lowland populations and two mountain populations were studied in two years. The study populations are near the centre of the species’ distribution range, where large populations occur locally (Hultén & Fries, 1986; Gustafsson & Sjögren-Gulve, 2002). *Gymnadenia odoratissima* produces nectar in a short floral spur, an inflorescence of 10-140 white to dark purple flowers, and a strong, sweet floral scent, which is important for pollinator attraction (Huber *et al.*, 2005). Floral traits differ considerably between lowland and mountain populations (Sun *et al.*, 2014; Gross *et al.*, 2016). A range of Lepidopteran families and genera are the major pollinators of *G. odoratissima,* but also other insect orders have been observed as pollinators, most importantly some Empididae (Diptera) at higher altitudes (van der Cingel, 1995; Huber *et al.*, 2005; Sun *et al.*, 2014; Gross *et al.*, 2016). The phenotypic data include three morphological traits (flower number, plant height, inflorescence length) and 22 floral volatiles. We analysed each dataset (i.e. each population-year combination) separately.

To assess and quantify the extent of spatiotemporal variation in selection we used the approach recently proposed by Albertsen *et al.* (2021), in which the among-dataset variation is computed as

$\sigma_{\beta}^{c}=\sqrt{\sigma_{\beta}^{2}-\bar{\mathrm{SE}_{\beta}^{2}}}$,

where σ^2^_β_ is the variance of the selection-gradient estimates among datasets, and SE^2^_β_ is the sampling variance of each selection-gradient estimate. In the current Bayesian framework, we used the variance of the posterior distribution as an estimate of the sampling variance (squared standard error). For mean-standardized selection gradients, this measure can be interpreted as the mean dispersion of the selection estimates in units of the strength of selection on fitness itself.

*Anacamptis coriophora*

Joffard et al. (2020) studied *Anacamptis coriophora*, a terrestrial, nectar-producing orchid species from central and southern Europe. *Anacamptis coriophora* comprises several subspecies, three of which were sampled in this study, namely *Anacamptis c. coriophora, A. c. fragrans* and *A. c. martrinii.* The first grows in low- to medium-altitude hay meadows and seasonally wet to wet grasslands, whereas the second grows in dry Mediterranean grasslands and the third in mountain hay meadows in the Pyrenees. These three subspecies produce inflorescences of about 10–30 flowers which open between May and July, have a light to dark pink colour and are strongly scented, with a stinkbug‐like smell for *A. c. coriophora* and a sweeter, vanilla‐like smell for *A. c. fragrans*. Three populations of *A. c. coriophora,* three populations of *A. c. fragrans* and one population of *A. c. martrinii*, all located in Southern France, were sampled in this study. In each population, about 50 individuals were sampled for floral colour and scent using calibrated photographs and dynamic headspace extraction, respectively.

The phenotypic data include three morphological traits (plant height, inflorescence length, number of flowers), and around 30 floral volatiles (with some variation among subspecies). We analysed each population separately.

*Penstemon digitalis*

Parachnowitsch *et al.* (2012) studied a common-garden population of *Penstemon digitalis* derived from three natural populations from Ithaca, NY (USA). These populations are old fields that were periodically mown to maintain herbaceous plants and reduce shrubs/trees. Other previous work has shown pollinator-mediated selection on flower size and display size in one of the source populations (Parachnowitsch & Kessler, 2010), however floral scent was not measured in that study. The phenotypic data include four morphological traits (flower size, daily display size, plant height, number of flowers), one phenological trait (flowering date), and two types of signalling traits: floral colour and floral scent. *Penstemon digitalis* has a white corolla with purple strips that vary in number and intensity, therefore colour was quantified as a composite trait of these two factors (Parachnowitsch *et al.*, 2012). The floral-scent emission data comprised 23 volatile concentrations.

**Extended comparison to Gross et al. (2016)**

Our *Gymnadenia odoratissima* results (see main text) are qualitatively comparable to those of Gross *et al.* (2016) in that selection on scent tended to be stronger in lowland populations, especially in the first year of study (see main text, Figure 1). Moreover, Gross *et al.* (2016) detected selection on scent primarily in the form of positive selection on the first (leading) principal component (PC1). Their PC1 was under stronger selection in the lowland than in the mountains. Seven compounds loaded strongly onto PC1, six of which were shown to be electrophysiologically active in pollinators (Huber *et al.*, 2005). Our results are consistent with these findings in that the same seven compounds were the ones under comparatively stronger positive selection and for most of them selection was stronger in the lowland than in the mountains (see main text, Figure 2). Together, this suggests good correspondence between our results and those of Gross *et al.* (2016) in terms of picking up populations and compounds under selection.

**References**

**Albertsen E, Opedal ØH, Bolstad GH, Perez-Barrales R, Hansen T, Pélabon C, Armbruster WS. 2021.** Using ecological context to interpret spatiotemporal variation in natural selection. *Evolution* **75**: 294-309.

**Chapurlat E, Ågren J, Anderson J, Friberg M, Sletvold N. 2019.** Conflicting selection on floral scent emission in the orchid *Gymnadenia conopsea*. *New Phytol* **222**: 2009-2022.

**Gross K, Sun M, Schiestl FP. 2016.** Why do floral perfumes become different? Region-specific selection on floral scent in a terrestrial orchid. *PLoS One* **11**: e0147975.

**Gustafsson S, Sjögren-Gulve P. 2002.** Genetic diversity in the rare orchid, *Gymnadenia odoratissima* and a comparison with the more common congener, *G. conopsea*. *Conservation Genetics* **3**: 225-234.

**Huber FK, Kaiser R, Sauter W, Schiestl FP. 2005.** Floral scent emission and pollinator attraction in two species of *Gymnadenia* (Orchidaceae). *Oecologia* **142**: 564-575.

**Hultén E, Fries M. 1986.** *Atlas of North European vascular plants: north of the Tropic of Cancer*. Königstein: Koeltz Scientific Books.

**Parachnowitsch AL, Kessler A. 2010.** Pollinators exert natural selection on flower size and floral display in *Penstemon digitalis*. *New Phytologist* **188**: 393-402.

**Parachnowitsch AL, Raguso RA, Kessler A. 2012.** Phenotypic selection to increase floral scent emission, but not flower size or colour in bee-pollinated *Penstemon digitalis*. *New Phytol* **195**: 667-675.

**Sletvold N, Trunschke J, Wimmergren C, Ågren J. 2012.** Separating selection by diurnal and nocturnal pollinators on floral display and spur length in *Gymnadenia conopsea*. *Ecology* **93**: 1880-1891.

**Sun M, Gross K, Schiestl FP. 2014.** Floral adaptation to local pollinator guilds in a terrestrial orchid. *Ann Bot* **113**: 289-300.

**van der Cingel NA. 1995.** *An atlas of orchid pollination. European orchids*. Rotterdam, Netherlands: Balkema.

**Øien DI, Moen A 2002.** Flowering and survival of *Dactylorhiza lapponica* and *Gymnadenia conopsea* in the Sølendet Nature Reserve, Central Norway. In: Kindlmann P, Willems JH, Whigham DF eds. *Trends and fluctuations and underlying mechanisms in terrestrial orchid populations*. Leiden, the Netherlands: Backhuys Publishers, 3– 22.
